# Well-being in Hemodialysis Patients

**Published:** 2018-08

**Authors:** Maryam SERAJI, Davoud SHOJAEIZADEH, Fatemeh RAKHSHANI

**Affiliations:** 1. Health Promotion Research Center, Zahedan University of Medical Sciences, Zahedan, Iran; 2. Dept. of Health Education & Health Promotion, School of Public Health, Tehran University of Medical Sciences, Tehran, Iran; 3. Dept. of Health Education & Health Promotion, School of Public Health, Shahid Beheshti University of Medical Sciences, Tehran, Iran

## Dear Editor-in-Chief

Nowadays, chronic kidney disease (CKD) has been increasing due to various factors (1). Due to lifetime increase, the number of these patients is increasing; but this disease influences their life and in its progressive stages, it can affect their performance and change their quality of life (2). Furthermore, the incidence of chronic kidney failure can lead to individual’s dependence on others, low self-esteem, and the feeling of loneliness, and it can affect the socio-mental aspect of an individual’s quality of life (3).

One of the issues presenting in hemodialysis patients is the well Being. Well-being is something greater than not being ill. That’s to say, having logic, independence, and self-confidence can be defined as well-being (4). It means that we accept an individual as a valuable person and know his or her feelings. Moreover, he or she is free to express the feelings such as anger, anxiety, fear, and pleasure (4). Well-being is a multidimensional procedure which includes mental, social, physical, and emotional health. This concept takes into account the positive attitude toward body (4). Kidney failure or end-stage renal disease (ESRD) is among the chronic diseases with great complications in which paying attention to welfare feeling and mental aspects of the affected patients is of great significance (5). One of the effective factors on well-being of hemodialysis patients is stress. The presence of stress in life can be attributed to various factors (6). One of the well-being aspects is social aspect: if a person feels well socially, he or she enjoys being with others and provides a positive relationship with others. This aspect of well-being includes social cooperation, social acceptance, and social integrity (4,7), another aspect the wellness is physical aspect which includes having a flexible, energetic, and strong body with a healthy heart (7). In addition, feeling well emotionally includes life satisfaction and positive effects on life (6). Another well-being aspect is spiritual aspect which has to do with the goals and meanings (sense) of life (8). Spiritual well-being includes honesty, forgiveness, hope, mercifulness, following a goal in life, and accepting comprehensive and unique concepts (8). There is a relationship between spiritual well-being and decrease of depression, increase of self-confidence, and decrease of disability (9).

Moreover, hemodialysis can have negative effects on general health and well-being of the patients; in addition, it can have negative influence on physical performance, mental status, and social relationships. Hemodialysis complications are not limited to physical and mental aspects, but they include economical aspect as well (2). Identifying well-being aspects including mental, social, physical, emotional, spiritual, and subjective aspects should be taken into consideration in order for the patients to be able to achieve their goals. In spite of considerable medical achievements and high prevalence of dialysis, death, and hospitalization rates during the last recent 20 years, no comprehensive study has been conducted on considering the well-being of hemodialysis patients in Iran. Therefore, the current study was designed and implemented to consider the well-being of hemodialysis patients referring to Zahedan University of medical sciences’ hospitals. Therefore this is a study on 129 hemodialysis patients referring to the hospitals of Zahedan University of medical sciences in 2016.

The present study showed that the mean score of well-being was more than average. Furthermore, the factors such as gender, educational level, and income level are known as the factors affecting emotional well-being in patients affected by Hemodialysis. Therefore, paying special attention to these patients’ need can lead to useful outcomes and will promote the patients’ life quality and lifespan. And health planners can also have useful and effective use of educational programs.
